# The State Children's Association

**Published:** 1910-11-26

**Authors:** 


### THE STATE CHILDREN'S ASSOCIATION.

The seventh report of the State Children's Association, covering the years 1907-08-09, shows a substantial advance on many points since the last was issued in 1907. The scope of the Association's work is constantly widening. Founded in 1896 to advocate the interests of Poor Law children in the Metropolis alone, it has of late years extended its operations to the provinces, and from only dealing with dependent children it has gone on to consider the case of young delinquents. The methods and organisation of the Association are by this time widely known. It continues as fortunate as ever in its officials, and in Lord Lytton and Sir Albert Spicer has secured an invaluable Chairman and Vice-Chairman. Events have of late marched rapidly, and proposals which three years ago seemed counsels of perfection are to-day established facts.

### The Children Act.

The Children Bill has become the Children Act. Before introducing it, Mr. Herbert Samuel asked the assistance of the Association, and suggestions were made with regard to the amendment of the Infant Life Protection Act, 1899, the necessity of legislation for the children of vagrant parents, the compulsory establishment of separate Courts of Justice for children, and the special treatment of feeble-minded delinquent children. It is gratifying to know that almost all the amendments to the Bill proposed by the Association were obtained. The amending of the Infant Life Protection Act is a special case for congratulation, for "one-child cases" are no longer exempt from protection, the age limit is raised from five to seven years, and foster-parents can no longer insure their helpless little charges. The result of these changes has been sufficiently striking. Last year in one Union alone the number of registered cases rose from ninety-two to 389, and the additional 297 were *all one-child cases*. Like evidence comes from other Unions, and to those who can read between the lines this provision, and the visits and advice of the protection visitors, means health and happiness and life itself to thousands of little children.

The Association has given its aid in promoting those courts of justice for children which the Children Act has made compulsory, and a special effort, attended by considerable success, was made to accelerate the working of the Probation of Offenders Act. A letter signed by Lord Lytton was addressed to all Chairmen of Petty Sessions suggested measures for ensuring its active working and for enlisting a large body of voluntary help on its behalf. Lord Lytton was invited to sit on the Departmental Committee which subsequently inquired into the working of the Act, and the S.C.A. was requested to furnish suggestions.

The Association is urging the compulsory appointment of women visitors and lady inspectors under the Infant Life Protection Act and the Order regulating boarding-out within the Union—a provision so eminently advisable that it is astonishing it should meet with any opposition.

The S.C.A. welcomes the corroboration of its principles given by the Poor Law Commission Reports both of the Majority and the Minority, which declare decisively against the extension of barrack schools, and pronounce against the maintenance of children in workhouses, an abuse which, often as it has been exposed, is still too common.

The Association is combating strongly the use of workhouses as places of detention for young offenders, and its Report points out that the Local Government Board has granted permission for their use for a limited period only, so that every effort must be made that this unsuitable arrangement shall not become permanent.

Boarding-out continues to increase in popularity, and excellent work has been done by the Association in forming fresh committees in neighbourhoods where none were formerly forthcoming.

In the Report of three years ago fifty-eight Unions were mentioned as having adopted the scattered home system of dealing with children ineligible for boarding-out. To-day that number has risen to nearly ninety, and to almost all of these the S.C.A. has been able to send information and suggestions, receiving letters of appreciation which show the hold obtained by the principles of the Association.

Provincial work grows apace, fresh boards of guardians are being drawn in, meetings are organised in increasing numbers, and leaflets, letters, and answers to all sorts of applications go out constantly from the Office.

Nothing so concerns a nation as the well-being of its children. The three years' record of the Association's work thoroughly justifies an appeal to the public for such support as will enable it to answer the calls to labour which reach it from many sides.

The Report can be obtained (price 4d. post free) from the offices of the Association, 53 Victoria Street, S.W.

